# What Is Time Good for in Working Memory?

**DOI:** 10.1177/0956797621996659

**Published:** 2021-07-26

**Authors:** Eda Mızrak, Klaus Oberauer

**Affiliations:** Department of Psychology, Cognitive Psychology Unit, University of Zürich

**Keywords:** working memory, time, encoding resource account, proactive benefit, memory, open data, open materials

## Abstract

Giving people more time to process information in working memory improves their performance on working memory tasks. It is often assumed that free time given after presentation of an item enables maintenance processes to counteract forgetting of this item, suggesting that time has a retroactive benefit. Two other hypotheses—short-term consolidation and temporal distinctiveness—entail a local effect of time on immediately preceding and following items. Here, we show instead a novel global and proactive benefit of time in working memory. In three serial-recall experiments (*N*s = 21, 25, and 26 young adults, respectively), we varied the position and duration of the free time within a seven-item list of consonants. Experiment 1 showed that the effect is global and not local. Experiments 2a and 2b showed that increased interitem time benefited performance only for the subsequent items, implying a proactive benefit. This finding rules out maintenance processes, short-term consolidation, and temporal distinctiveness as explanations of the free-time benefit but is consistent with the proposal of a gradually recovering encoding resource.

In the context of short-term or working memory, the passage of time is usually thought of as an opportunity to forget (Donkin et al., 2015; Lewandowsky & Oberauer, 2009; Mercer & McKeown, 2014; Ricker et al., 2016, 2020). A less well-studied role of time is that, under some circumstances, it helps maintain information in working memory. When a memory list is presented more slowly—that is, with more free time between items—immediate serial recall is often found to be better (Ricker & Hardman, 2017; Souza & Oberauer, 2017; Tan & Ward, 2008; for reviews, see Oberauer et al., 2018; Penney, 1975). Here, we asked what causes this beneficial effect of time for working memory.

One possible explanation is that free time between items is used for rehearsal. Rehearsal is a commonly reported maintenance strategy in working memory tasks. Three forms of rehearsal could contribute to the beneficial effect of free time: articulatory rehearsal (Tan & Ward, 2008), attention-based refreshing (Barrouillet & Camos, 2015), and elaborative rehearsal (Bartsch et al., 2018). In articulatory rehearsal, to-be-remembered information is repeated verbally during its maintenance. In attention-based refreshing, information is reactivated by deliberately attending to it during maintenance. In elaborative rehearsal, representations of to-be-remembered stimuli are enriched by associating them with long-term memory knowledge.

Free interitem time can also be used for short-term consolidation (Jolicœur & Dell’Acqua, 1998) of the just-encoded item. Short-term consolidation takes place after encoding of an item; it is estimated to take about 0.5 s to 1.5 s and assumed to require a central processing resource (Jolicœur & Dell’Acqua, 1998; Nieuwenstein & Wyble, 2014).

A third explanation comes from the temporal-distinctiveness hypothesis. According to temporal-distinctiveness theories of memory, increasing the time between items decreases the similarity of their temporal contexts, which in turn should decrease temporal confusability and increase memory accuracy (Brown et al., 2007). A related idea is that exceptionally long interitem times—for instance, when subsets of list items are temporally grouped (Ryan, 1969b)—induce a context shift, increasing the contextual distinctiveness between items in different groups.

These explanations lead to different predictions about which items in a memory list benefit from increased free time. We consider differences of predictions along two dimensions (see Table 1), which can best be explained by focusing on a single interitem interval somewhere in the middle of a memory list: (a) The beneficial effect of free time in that interval can be retroactive (i.e., improving memory for items encoded before the interval) or proactive (i.e., improving memory for subsequently encoded items), and (b) the beneficial effect can be local (i.e., improving memory only for the items immediately preceding or following the free-time interval) or global (i.e., improving memory for all list items preceding or following the interval).

**Table 1.** Summary of the Predictions From Different Mechanisms That Can Be Applied During Free Time and Accounts That Predict How Free Time Affects Memory

Effect	Proactive	Retroactive	Proactive and retroactive
Global		Cumulative articulatory rehearsal, cumulative refreshing, elaboration of sets of items	Context shift, temporal grouping
Local	Short-term consolidation (interrupted)	Short-term consolidation (ballistic), elaboration of single items	

The three forms of rehearsal (articulatory rehearsal, elaborative rehearsal, and attentional refreshing) can be applied only to items already encoded into working memory before a free-time interval, and therefore, their effect must be primarily retroactive. Articulatory rehearsal is usually cumulative, and therefore, the effect should be retroactive and global, benefiting all items encoded before the free-time interval used for rehearsal. Refreshing is also commonly assumed to cycle through all items in working memory, rather than to dwell on the last-presented item, implying a global retroactive effect (Barrouillet et al., 2007; Lemaire et al., 2018; Oberauer & Lewandowsky, 2011). In contrast, elaboration could involve all items encoded so far or only the last-encoded item, so the effect could be global or local.

Memory enhancement for items preceding a free-time interval could have indirect effects on the subsequent items as well. For instance, if free time is used to improve maintenance of previously encoded items, then when enough time is given, maintenance processes such as rehearsal or refreshing of these items can be completed during that time. This could diminish the cost of rehearsing or refreshing preceding items during encoding or maintenance of the subsequent items and thereby improve maintenance of the subsequent items. In this case, a proactive effect could occur in addition to the retroactive effect.

**Statement of Relevance**

Working memory is our mind’s blackboard, where we can keep information available briefly—for instance, we can hold a new phone number in working memory and then type it from memory. The passage of time is usually associated with forgetting of information held in working memory: Many researchers believe that information in working memory fades quickly unless we rehearse it by repeating it to ourselves. In contrast to this idea, research shows that if we pause in between adding items to working memory, our memory improves. We investigated what people use these pauses for. For instance, one could use a pause to go over what is already in working memory (e.g., rehearsal). Instead, we found that pauses improve recall of information that is added to working memory *after* the pause without leading to any forgetting of items already in working memory before the pause. This finding suggests that pauses (i.e., time) help working memory prepare for future information and calls for a new way of thinking about the role of time in working memory.

Short-term consolidation is commonly assumed to apply only to the last-encoded item. Moreover, it relies on a limited processing resource, so that most theorists assume that only one item is consolidated at any time (for a review, see Ricker et al., 2018). This conceptualization suggests that in any free-time interval, only the immediately preceding item is consolidated. If each item is consolidated only until interrupted by the onset of the next item, the beneficial effect of free time has to be retroactive and local: Longer free time enables longer consolidation of the one preceding item. Ricker and Hardman (2017) have proposed an alternative hypothesis: Short-term consolidation is a ballistic process that, once started, runs to completion. When not enough time is given for consolidation to be completed, consolidation of the next item is postponed and thereby curtailed (Ricker & Hardman, 2017). Increased free time avoids that postponement and thereby improves memory for the subsequent item, predicting a local proactive benefit only for this item. In a series of visual working memory experiments, Ricker and Hardman (2017) obtained evidence for such a local, proactive effect.

According to the temporal-distinctiveness hypothesis, longer interitem free time should increase the temporal distinctiveness of the items immediately before and after the free-time interval (Brown et al., 2007). Hence, temporal distinctiveness predicts local effects that are both proactive and retroactive. This prediction has been tested in several studies. Whereas the predicted effects have been observed in recognition tests (Morin et al., 2010) and some versions of reconstruction-of-order tests, they are conspicuously absent in immediate serial-recall tests (Lewandowsky et al., 2006; Nimmo & Lewandowsky, 2005, 2006; Parmentier et al., 2006; Peteranderl & Oberauer, 2018).

Similarly, context shifts between temporal groups predict symmetric proactive and retroactive benefits that are predominantly local but also to some extent global (Burgess & Hitch, 1999; Farrell, 2012). Such effects are commonly observed in serial recall, leading to within-group primacy and recency effects (Frankish, 1989; Ryan, 1969a).

To understand what free time is used for in working memory, we tested (a) whether the free time has local or global effects and (b) whether the effect of free time is proactive, retroactive, or both. Experiment 1 focused on adjudicating between global and local effects. We varied the durations of interitem time within the lists. Interitem times were either consistently short across the list, consistently long, or varied within a list differently for all positions, such that the average interitem time was as long as in the consistently long condition. The aim of the variable-interval condition was to test whether the duration of each interitem interval has an effect predominantly on the adjacent items (i.e., local effects) or spreads across the list items (i.e., a global effect).

The variable-interval manipulation replicates the design of Lewandowsky et al. (2006) for testing the temporal-distinctiveness hypothesis. Serial-recall studies with this design found no evidence for local effects of time, which contradicts the predictions of temporal distinctiveness. One possibility that we need to consider, however, is that people use free interitem intervals for processes such as elaborative rehearsal or short-term consolidation only if their duration is predictable. In that case, the unpredictably varying intervals in the temporal-distinctiveness studies might not have been used for any process improving memory. If so, memory in the variable-interval condition should be poorer than in the condition with consistently long intervals, despite providing the same amount of free interitem time overall.

With Experiments 2a and 2b, we tested to what extent the free-time benefit was proactive or retroactive. We increased only one interitem time, whereas the rest were fixed. The position of the longer interitem time was varied throughout the list. The longer interitem interval could be 2,500 ms or 500 ms, whereas the regular interitem intervals were 50 ms each. We asked whether the long interval had an effect for the preceding items (retroactive), the following items (proactive), or both. In addition, we expected to see temporal-grouping effects due to the deviant interitem interval for both 500-ms and 2,500-ms intervals. Because Ryan (1969b) observed no difference in grouping effects between short and long intergroup intervals, we predicted these grouping effects to be equivalent for both deviant lengths. The aim was to observe whether the extra free time (2,000 ms) given at different positions in the memory list would benefit performance for the items observed before or after the manipulated interval, over and above temporal-grouping effects.

## Method

### Participants

Twenty-one, 25, and 26 young adults participated in Experiments 1, 2a, and 2b, respectively. Sample sizes were chosen on the basis of previous experiments that have shown beneficial effects of longer interitem intervals. Data collection was stopped when we reached a prespecified target sample size (*N* + 1, if possible, in case we needed to exclude any data during analysis). Experiment 1 had a target sample size of 20, and Experiments 2a and 2b had a target sample size of 25. Experiments lasted up to 60 min. Participants were reimbursed for their time with a course credit or 15 Swiss francs per hour.

### Procedure

**Fig. 1.**
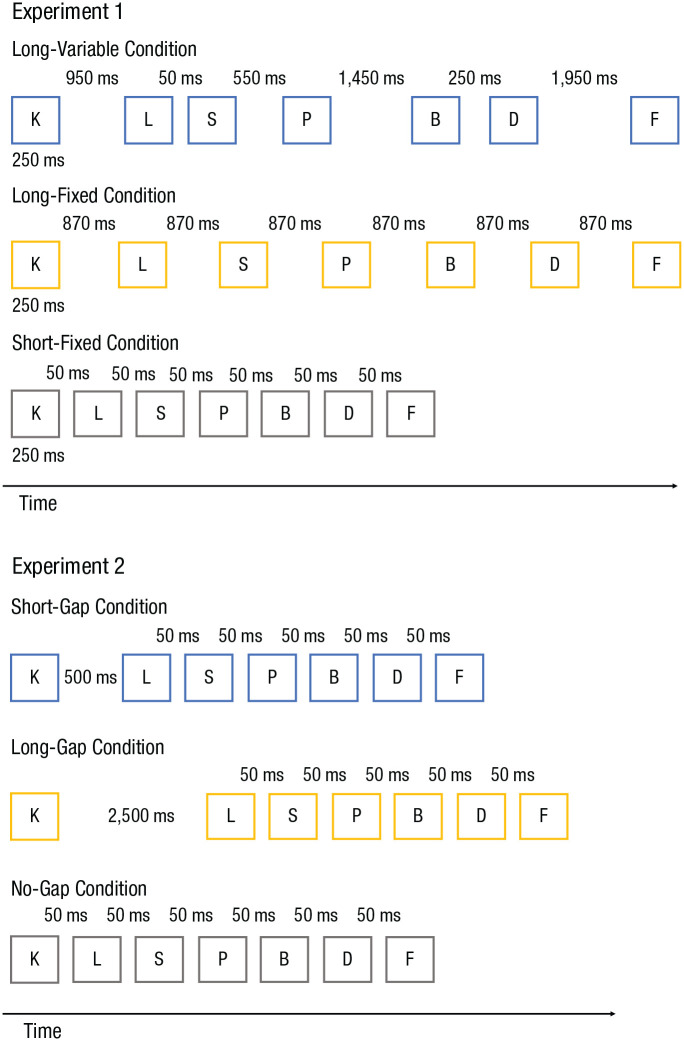
Timeline of the encoding phase in each of the conditions from Experiments 1 and 2. In each condition, seven consonants, randomly drawn from 21 consonants, were presented one at a time. This was followed by a serial-recall test. Interstimulus intervals (ISIs), defined as the total interval from offset of one consonant to onset of the next one, varied across conditions. The numbers above the lists indicate the ISIs for the consonants beneath them. In Experiment 1, the last consonant was always presented for 250 ms and was followed by the retention interval. The retention interval, the time between the offset of the last consonant and the test, was fixed for all conditions (1,250 ms). In the long-variable condition, six different ISIs were randomly assigned to each ISI position within a list (50, 250, 550, 950, 1,450, and 1,950 ms). In the long-fixed condition, ISIs were fixed (870 ms); the sum of the ISIs was approximately the same as in the long-variable condition. In the short-fixed condition, ISIs were fixed (50 ms), and their sum was shorter than in the other conditions. In Experiment 2, one of the ISIs could be longer than the remaining ISIs, introducing a gap in the encoding phase and providing free time between study items. This gap was 500 ms in the short-gap condition and 2,500 ms in the long-gap condition. In the examples of the short- and long-gap conditions shown here, the gap appears after the first item in the study list. In the actual experiment, the gap could be in any position in the list. All other ISIs in Experiment 2 (including those in the no-gap [baseline] condition) were 50 ms. Only Experiment 2b had the no-gap condition.

Each trial began with a central fixation point presented for 500 ms, followed by presentation of the study list. Lists consisted of seven consonants presented one at a time (see Fig. 1). In Experiment 1, each list item was presented on screen for 250 ms, followed by a blank screen for the remainder of the interstimulus interval (ISI), here defined as the total interval from offset of one consonant to onset of the next one. In Experiments 2a and 2b, each list item was presented for 300 ms followed by a blank screen for the remainder of the ISI. The standard ISI in Experiments 2a and 2b was 50 ms. In all experiments, list presentation was followed by a delay (1,250 ms for Experiment 1; 1,000 ms for Experiments 2a and 2b), and then participants started the immediate serial-recall test. Participants were instructed to type the letters in their order of presentation. They had to enter seven items before they could proceed to the next trial.

#### Experiment 1

The experiment consisted of six blocks of 18 trials each, resulting in 108 trials. To test whether the free-time effect was global or local, we manipulated the duration of ISIs. An ISI is the total interval from offset of one item to the onset of the next item. There were three conditions: a *short-fixed* condition consisting of short ISIs (50 ms) across the list, a *long-fixed* condition consisting of longer ISIs (870 ms) across the list, and a *long-variable* condition consisting of variable ISIs. Each participant received an equal number of trials in each of these conditions, in random order.

The key manipulation was the long-variable condition, which followed the design of Lewandowsky et al. (2006). There were six different ISIs in this condition: 50 ms, 250 ms, 550 ms, 950 ms, 1,450 ms, and 1,950 ms. In each trial of the long-variable condition, each of these ISIs was assigned to one interitem position in the list. There were 720 possible orders of six intervals; these orders were assigned to the 20 participants by an algorithm that minimized the variability in the frequencies of using each order. In this way, the ISI preceding or following each item was unconfounded with the item’s serial position. The sum of ISIs in the long-variable condition (5,200 ms) was approximately equal to the sum of ISIs in the long-fixed condition (5,220 ms). The time after the last item in the lists was fixed for all conditions (1,250 ms).

#### Experiment 2a

The experiment consisted of eight blocks of 36 trials each, resulting in 288 trials. In each trial, there was a deviant ISI at one interitem position, either short (500 ms) or long (2,500 ms). Both created a temporal gap in contrast with the background of the remaining standard ISIs, which were all 50 ms. Such a gap is known to give rise to temporal grouping (Ryan, 1969b), but because Ryan (1969a) has shown equivalent grouping effects for short and long gaps, we expected no difference in grouping effects between the short-gap and the long-gap conditions. We investigated whether, on top of the common grouping benefit, the extra free time given in the long-gap condition improves memory for items preceding free time or items subsequent to free time. There were six positions in the study list where the gap could be inserted: following any item from the first through the sixth. In total, there were 12 conditions: six gap positions by two gap durations. Each block consisted of three trials of each condition, resulting in 24 trials per condition.

#### Experiment 2b

Experiment 2 was the same as Experiment 1, except for one difference. We added a baseline condition in which there were no gaps, in order to examine general effects of the gap, such as temporal grouping. In the baseline condition, there was no deviant ISI; all ISIs were 50 ms. This made a total of 13 conditions. Each block consisted of three trials of each condition, resulting in 312 trials overall.

### Materials

For each list, seven consonants were randomly drawn without replacement from the 21 consonants of the German alphabet.

### Data analysis

We estimated Bayesian linear mixed-effects models using the lmBF function from the *BayesFactor* package (Version 0.9.12-4.2; Morey & Rouder, 2018) implemented in the R programming environment (Version 4.0.1; R Core Team, 2020). Our analysis followed a model-selection approach focusing only on the set of “plausible models” implied by the principle of marginality (Rouder et al., 2016). More specifically, for each experiment, we estimated the full set of plausible models and then compared all of the models with the null model, which contained only an intercept and a random effect of subjects, using Bayes factors (BFs). The model with the largest BF was used to determine which of the effects (i.e., main effects and interaction) the data provided evidence for or against. Because our data contained repeated measures (for all factors in all experiments), we performed this step twice—once for the minimal model in which the random-effects structure contained only random intercepts and once for the maximal random-effects structure justified by the design (Barr et al., 2013). Below, we report results based on the maximal model. Unless otherwise stated, the pattern of BFs (i.e., providing evidence for or against a specific effect) was the same for the set of models using the minimal random-effects structure. Full results are also provided in the Supplemental Material available online. All analyses were performed on the data aggregated by participant and cell of the design. Therefore, the maximal random-effects structure justified by the design did not entail random slopes for the highest order effect (e.g., highest order interaction; Singmann & Kellen, 2020).

Results reported below are often given in the form of BF_10_, indicating the strength of evidence for a particular focal model, Model 1, against a comparison model, Model 0. The value of BF_10_ indicates how much more likely Model 1 is over Model 0. If the value of BF_10_ is greater than 1, this indicates evidence for the alternative model (i.e., Model 1 over Model 0). If the value of BF_10_ is less than 1, this indicates evidence for the simpler model (i.e., Model 0 over Model 1). In the latter case, we report BF_01_ instead, which is given by BF_01_ = 1/BF_10_ so that BF_01_ values larger than 1 indicate evidence for the simpler model. BFs cannot be interpreted as *p* values and do not provide a cutoff for significance. A larger BF indicates stronger evidence for the winning model. As an interpretative guideline, BFs smaller than 3 are considered weak evidence, BFs between 3 and 10 are considered substantial evidence, and BFs larger than 10 or smaller than 0.1 are considered strong evidence (Kass & Raftery, 1995).

For Experiment 1, the first analysis included two factors, serial position and condition, and the second analysis included only one factor, ISI, which required comparison of only one Model 1 over Model 0. For Experiments 2a and 2b, the analysis included three factors, which resulted in several plausible models that could explain the data. For the analyses that included more than one factor, we first examined the BF_10_ for each model in comparison with the null model and found the model describing the data with the strongest evidence shown by the BF_10_ value. The model with the highest BF_10_ was then compared with additional models for testing specific hypotheses about the presence or absence of individual effects. This can be done by simply dividing a BF_10_ of the model including the effect with a BF_10_ of the model excluding the effect, which provides a BF in favor of the effect. The null models from these BF_10_ values must be the same for the new BF to be meaningful. In some cases, follow-up analyses also employed Bayesian *t* tests. In all the experiments, performance refers to serial-recall accuracy, which assigns a correct response to each list item only if that item was recalled in the correct output position.

## Results

### Experiment 1

**Fig. 2.**
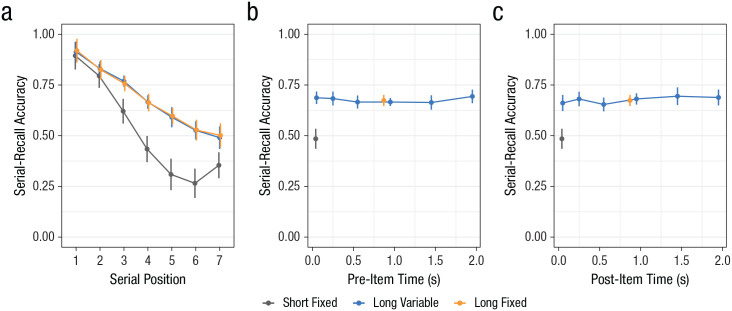
Proportion of accurate responses on the immediate serial-recall task in the three conditions of Experiment 1. The graphs show (a) average performance for each condition across serial positions, (b) performance for each condition averaged across serial positions as a function of pre-item time, and (c) performance for each condition averaged across serial positions as a function of post-item time. Serial-recall accuracy was determined by assigning a correct response to each list item only if that item was recalled in the correct output position. Error bars denote 95% within-subjects confidence intervals.

Our aim in Experiment 1 was to examine local and global effects of free time in working memory. The model comparison showed strong evidence for the full model. There was an interaction between condition (short fixed, long fixed, and long variable) and serial position (BF_10_ > 10,000 compared with both the null model and the second-best model, which consisted of both main effects). As can be seen from Figure 2a, performance in the long-fixed and long-variable conditions was better than performance in the short-fixed condition (both BF_10_s > 10,000 from Bayesian *t* tests comparing performance between conditions aggregated across serial position). Furthermore, performance in the long-fixed and long-variable conditions did not differ (BF_01_ = 7.7, implying evidence for the null model of no difference between the two conditions).

Second, we analyzed the long-variable condition, looking in detail at how pre-item and post-item time—the ISIs immediately preceding or following an item—affected memory of each item. For this analysis, Serial Positions 1 and 7 were excluded, because Serial Position 1 did not have a pre-item time, and Serial Position 7 did not have a post-item time. The fixed effects for the second analysis were (a) the duration of pre-item time and (b) the duration of post-item time. Both pre-item time and post-item time varied between 0.3 s and 2.2 s, and there were six durations. If free interitem time has a local effect, then performance should improve with longer pre-item time, longer post-item time, or both. If free interitem time has a global effect, no such effect would be predicted because the total free interitem time was constant for all trials of the long-variable condition.

We found no effect of pre-item or post-item time duration on performance (see Figs. 2b and 2c). There was strong evidence against both pre-item time (BF_01_ > 10,000) and post-item time (BF_01_ > 10,000), ruling out any local effect of time.

### Experiments 2a and 2b

Our aim in Experiments 2a and 2b was to test whether the free-time benefit is proactive, retroactive, or both. Additionally, the free-time benefit could be local or global. To test these possibilities, we focused on the effect of free time as a function of the lag between the gap and the presented items. By doing so, we could analyze the impact of free time on preceding and subsequent items separately. The lag was calculated as the signed distance of an item from the position of the gap in the list. For instance, if the gap was between the third and fourth items, the third item would be at Lag −1 and the fourth item would be at Lag +1. Accordingly, there were 10 lags: −5, −4, −3, −2, −1, +1, +2, +3, +4, and +5. Negative lags included items preceding the gap and were used to test retroactive effects. Positive lags included items subsequent to the gap and were used to test proactive effects. For example, a lag of +2 would include (a) an item at Serial Position 4 if the gap was between Items 2 and 3, (b) an item at Serial Position 5 if the gap was between Items 3 and 4, and (c) an item at Serial Position 6 if the gap was between Items 4 and 5. Memory performance for Lag +2 would then be calculated by averaging across serial-recall performance for these items in the appropriate gap-position conditions.

To examine the proactive and retroactive effects of free time on memory performance, we tested the interactions of added free time in the gap (450 ms vs. 2,450 ms) with the sign of the lag and its absolute value. The sign of the lag indicated whether an item preceded (negative lag) or followed (positive lag) the manipulated interval, and therefore, the interaction of free-time duration with lag sign told us whether the effect of free time was more retroactive or more proactive. The interaction of free-time duration with absolute value of the lag—in particular, the contrast between Lags ±1 and larger absolute lags—told us whether the effect was local or global.^1^

**Fig. 3.**
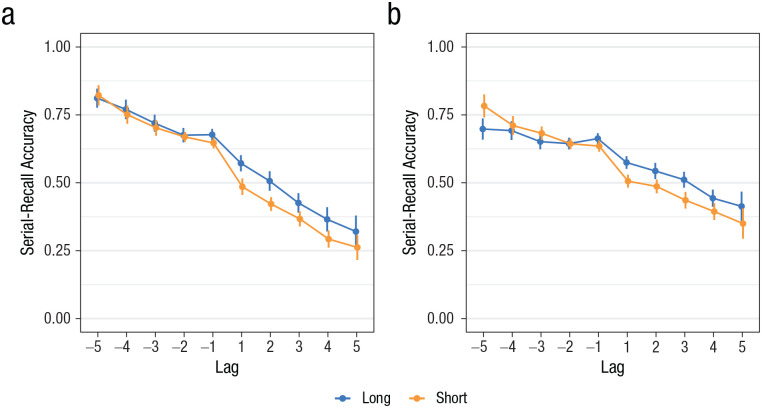
Proportion of accurate responses on the immediate serial-recall task for preceding and subsequent items as a function of lag (−5 to 5) and amount of free time (long, short) in Experiments 2a (a) and 2b (b). Serial-recall accuracy was determined by assigning a correct response to each list item only if that item was recalled in the correct output position. Error bars denote 95% within-subjects confidence intervals.

Figure 3 presents performance across conditions in Experiments 2a and 2b. There was a notable proactive effect on memory; performance was better for the items following a long gap compared with the items following a short gap. There was no difference between the effect of long and short free time on performance for the preceding items. In other words, there was no retroactive effect of gap duration.

For Experiment 2a, the best model included main effects of free time, lag sign, and absolute lag, as well as the free-time-by-lag-sign interaction, but no interactions involving free time and absolute lag (BF = 37 compared with the full model, which included all two-way interactions and the three-way interaction, and BF = 3.7 compared with the second-best model, which included all two-way interactions but not the three-way interaction). For Experiment 2b, the best model was the full model (BF = 495 compared with the second-best model with no three-way interaction).

To dissect the interaction of free-time duration with lag sign, we examined the pairwise comparisons between long and short free time separately for subsequent items (positive lags) and preceding items (negative lags) with Bayesian *t* tests. In both Experiments 2a and 2b, extra free time in the long-free-time condition improved performance for subsequent items compared with the short-free-time condition, providing strong evidence for a proactive benefit (Experiment 2a: BF_10_ = 1,137; Experiment 2b: BF_10_ = 885). In contrast, the evidence for retroactive benefits was rather weak. In Experiment 2a, extra free time improved performance for preceding items by only a small amount (ambiguous evidence for a retroactive benefit; BF_10_ = 1.15). In Experiment 2b, there was no evidence for a retroactive benefit of extra free time, and instead, there was weak evidence against such a benefit (BF_01_ = 2.6).

Our next analysis focused on whether the free-time effect changes with the absolute lag. Any local effect would be signaled by an interaction of free time with absolute lag. We zoomed in on this interaction separately for preceding and subsequent items.

The results from Experiment 2a did not indicate any interaction of free time and absolute lag for both preceding and subsequent items. For subsequent items, the best model included only the two main effects (BF compared with the full model = 6). For preceding items, the best model included only a main effect of absolute lag; however, the evidence favoring this model over the second-best model, which included the two main effects, was ambiguous (BF_10_ = 1.17).

The results from Experiment 2b provided evidence for an interaction between absolute lag and free time only for the preceding items and not for the subsequent items. For the subsequent items, the best model included only the two main effects (BF compared with the full model = 9.53). For the preceding items, there was evidence for an interaction between the absolute lag and free time (BF_10_ compared with the second-best model > 1,000). The interaction appears to be driven by the absolute Lag 5 for preceding items, which is Lag −5 in Figure 3b. As can be seen from Figure 3b, at Lag −5, performance was lower for long free time compared with short free time. This effect is the opposite of a free-time benefit and therefore does not support the assumption of a retroactive benefit of time.

### Temporal-grouping effects

Experiments 2a and 2b were designed to examine the free-time benefit by giving extra free time in one of the ISIs. A temporal gap at one ISI is known to introduce temporal grouping, and therefore, we need to distinguish the free-time effect from the grouping effect. We assumed that both the short gap and the long gap induced grouping to the same degree, so that any additional effect of a long gap versus a short gap reflects the effect of free time. Here, we provide evidence for this conjecture.

Temporal-grouping effects are typically characterized by a sharp increase in the interresponse times for recalling the item following the gap and an increase in serial-recall performance both before and after the gap (Farrell et al., 2011). To check whether equivalent temporal grouping was induced for both the short- and long-gap conditions, we examined the data from Experiment 2b for commonly observed temporal-grouping effects on serial recall. We chose Experiment 2b because a baseline condition was included in this experiment, which could serve as a control list.

**Fig. 4.**
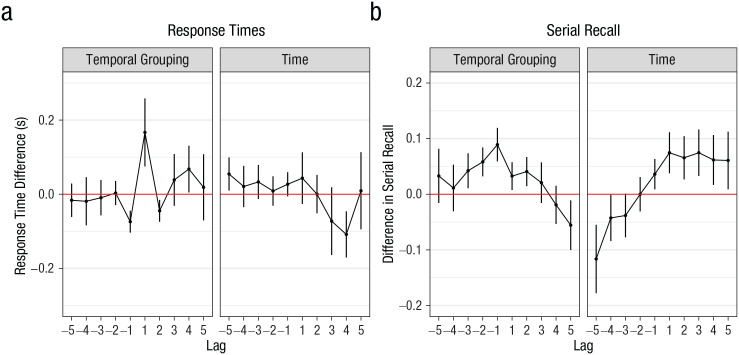
Effects of temporal grouping and time on (a) response times and (b) serial recall in Experiment 2b. The temporal-grouping effect was determined by calculating the difference in performance between the short-gap and no-gap conditions, and the time effect was determined by calculating the difference in performance between the long-gap and short-gap conditions. The red line at 0 indicates no difference between conditions. Serial-recall accuracy was determined by assigning a correct response to each list item only if that item was recalled in the correct output position; the proportion of such correct responses was used as a marker of accuracy. Differences in both response time and serial-recall performance are shown as a function of lag. Error bars denote 95% within-subjects confidence intervals.

We computed the difference of interresponse times at recall, and of serial-recall accuracy, between the three conditions in two steps. First, we subtracted performance (i.e., interresponse times and accuracy) of the no-gap condition from the short-gap condition. This difference should mainly reflect temporal-grouping effects. Second, we subtracted performance in the short-gap condition from performance in the long-gap condition. This difference should reflect the time effect over and above the effect of grouping. These differences are plotted as a function of lag in Figure 4. Figure 4 shows that the time effect was qualitatively different from the temporal-grouping effect. Whereas grouping selectively increased interresponse times to the Lag +1 item (i.e., the item following the gap), the time effect did not. Moreover, the temporal-grouping effect on serial-recall accuracy was symmetric—both sides of the gap improved because of temporal grouping—whereas the time effect was asymmetric, benefiting only items following the gap (for statistical support for these observations, see the Supplemental Material). In conclusion, the effects of grouping and of extended free time are qualitatively different, demonstrating that the time effect is not just an amplified effect of grouping.

### Summary

These results collectively indicate that (a) free time improves memory for subsequent items and not for the preceding items, indicating a purely proactive benefit, and (b) the proactive-free-time benefit does not interact with the absolute lag, indicating a global effect. The latter finding is important because the assumption of ballistic short-term consolidation (Ricker & Hardman, 2017) predicts such a proactive effect only for Lag +1 and not for other lags. In the present experiments, the proactive effect was not specific to Lag 1 and, therefore, cannot be explained by short-term consolidation.

## Discussion

We showed that free time has a global and proactive effect on immediate serial-recall performance. Results from the first experiment showed that the free-time effect is not local but global, as indicated by a benefit of free time spreading across the list items. The second experiment and its replication provided evidence for a purely proactive benefit. The purely proactive nature of the time benefit in working memory is consistent with the finding that additional time is helpful only between presentation of items but not after presentation of the entire list (Oberauer & Lewandowsky, 2016).

These findings cannot be explained by rehearsal or by short-term consolidation, which assume that free time can be used for strengthening representations of preceding items. Our findings imply either that extra free time (on top of 250–300 ms presentation time) was not used for these processes or that these processes were not helpful.^2^

Our findings also cannot be explained by temporal distinctiveness or by a context shift. Temporal distinctiveness predicts local benefits that are symmetrically proactive and retroactive, contrary to what we found. In Experiment 2, the deviant temporal gap arguably induced a shift to a new group context (Burgess & Hitch, 1999; Farrell, 2012). Perhaps a longer gap induced stronger grouping? Against this, we found that the empirical signature of grouping was qualitatively different from that of the free-time benefit (see Fig. 4).

Perhaps the model of Farrell (2012) could provide a grouping-based explanation for why the long gap benefited only the postgap items. In this model, the last group enjoys particularly high accessibility because its context is still active at the end of the list. However, because our task was serial recall, participants had to start recalling the first group first, which requires reinstating the first group context, at which point the last group loses its benefit. Therefore, Farrell’s model of grouping cannot explain the present findings.

Because our results do not agree with any established theoretical proposal, we asked how we could explain them. One possible explanation is that free time enables ad hoc chunking of the preceding items or outsourcing them into long-term memory (or both), thereby reducing the load on working memory, which facilitates maintenance of subsequent items. This explanation would raise the question of why these processes leave memory for the preceding items unchanged. Chunking is usually accompanied by substantially improved memory for the chunked information (Chen & Cowan, 2005; Miller, 1956; Thalmann et al., 2019). Outsourcing information into long-term memory could be expected to reduce accuracy because information in long-term memory is vulnerable to proactive interference building up across trials. It would be an unlikely accident if such transformations of the representations of early list items left their accessibility unchanged.

Alternatively, a recent theory by Popov and Reder (2020) proposed that there is a limited resource for encoding information into episodic memory that depletes with each item encoded, and this resource gradually recovers over time. If we transfer that idea to the domain of working memory, it could explain the findings of our study: (a) Each trial starts with a limited encoding resource, (b) each encoding event takes a fixed proportion of the available resources, and (c) during each interitem interval, the resource recovers gradually. It follows that the resource recovers more with longer ISIs. This benefit occurs only for items following the ISI, leading to a purely proactive benefit. The benefit is global because each item takes a constant proportion of the available resource. After the resource is replenished during a long interval, that proportion is a larger amount for all subsequent items.^3^

**Fig. 5.**
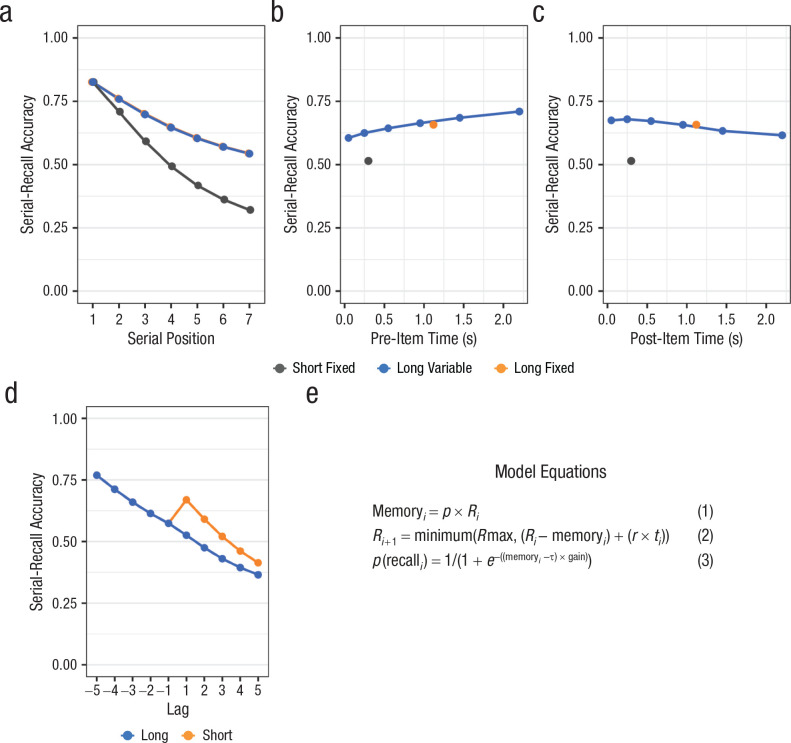
Simulated data for Experiment 1 (a, b, c) and Experiment 2 (d) along with model equations (e). The graphs show (a) average performance for each condition across serial positions, (b) performance for each condition averaged across serial positions as a function of pre-item time, (c) performance for each condition averaged across serial positions as a function of post-item time, and (d) performance on the immediate serial-recall task for preceding and subsequent items as a function of lag and amount of free time. In all graphs, serial-recall accuracy was determined by assigning a correct response to each list item only if that item was recalled in the correct output position; the proportion of such correct responses was used as a marker of accuracy. The data were generated with a model that simulates how a limited encoding resource evolves during encoding of a list. Each trial starts with a maximum amount of resource, *R*max, and each item, *i*, consumes a constant proportion *p* of the available resource *R_i_* for being encoded. The resource amount Memory_*i*_ assigned to the item determines its memory strength (Equation 1). During interitem time *t_i_* following item *i*, the resource is replenished with a constant rate *r*, up to the maximum *R*max (Equation 2). Probability of recall was calculated with a logistic function of the memory strength based on Equation 1, with τ and gain parameters. Thus, the model has four parameters: first, *p*, the proportion of resource that each item consumes from the available pool of resources, between 0 and 1; second, *r*, the rate at which the resource is replenished per second—this determines the increase in resources with free time; third, gain; and fourth, τ, jointly determining the conversion of memory strength to probability of recall. Parameter values used for this simulation are *p* = .23, *r* = .11, gain = 13, and τ = 0.11.

The encoding-resource account is a novel idea and therefore has not been applied to immediate recall yet. We built a simple model incorporating the encoding-resource idea to see whether the observed data patterns in our experiments can be predicted by it.^4^ Figure 5 shows simulated data for Experiments 1 and 2, together with the model equations and descriptions. The model predicts equivalent performance for long-fixed and long-variable conditions for Experiment 1, in line with our finding, and also the interaction of condition with serial position. However, the model also predicts slightly lower performance for shorter pre-item times, which we did not observe. The model accurately reproduced the global and proactive time benefit in Experiment 2.

We explored whether the model can also accommodate the local proactive benefit of free time that Ricker and Hardman (2017) observed for visual stimuli. We found that, with faster resource depletion and faster replenishment, this was the case (see Fig. S2 in the Supplemental Material). Therefore, the encoding-resource assumption implies a proactive effect, which—depending on model parameters—can be either more global or more local.

In summary, in three experiments, we documented a novel beneficial effect of free time on working memory that is proactive and global. Maintenance processes that could take place during free time predict a retroactive benefit, whereas the short-term-consolidation account as well as the temporal-distinctiveness hypothesis predict a local benefit. A context shift, as envisioned in some models of temporal grouping, predicts symmetric proactive and retroactive benefits. Therefore, the current findings cannot be explained by maintenance, consolidation, temporal-distinctiveness, or context-shift accounts in their current form (for predictions of these accounts, see Table 1). At present, only a novel encoding-resource account offers a promising explanation of the current findings. The novel empirical findings here support the possibility of working memory being subject to a limitation of an encoding resource that depletes with each item being encoded and recovers with time.

## Supplemental Material

sj-docx-1-pss-10.1177_0956797621996659 – Supplemental material for What Is Time Good for in Working Memory? Supplemental material, sj-docx-1-pss-10.1177_0956797621996659 for What Is Time Good for in Working Memory? by Eda Mızrak and Klaus Oberauer in Psychological Science

## Transparency

*Action Editor:* Daniela Schiller

*Editor:* Patricia J. Bauer

*Author Contributions*

Both authors designed and programmed the experiments and collected the data. E. Mızrak analyzed the data and drafted the initial manuscript, and K. Oberauer provided critical feedback and revisions. Both authors approved the final manuscript for submission.

**Open Practices:** All data have been made publicly available via OSF and can be accessed at https://osf.io/egz64. The design and analysis plans for the experiments were not preregistered. This article has received the badges for Open Data and Open Materials. More information about the Open Practices badges can be found at http://www.psychologicalscience.org/publications/badges.

## Notes

1. Given that the first item of a study list could not be a subsequent item, and the last item of a study list could not be a preceding item, we excluded these items from the analysis. Therefore, we excluded Absolute Lag 6, which included only the first or the last item.

2. Our findings do not rule out a contribution of short-term consolidation over shorter time scales: Ricker and Hardman (2017) have shown a local proactive benefit of free interitem intervals up to about 500 ms. Such rapid consolidation would contribute little to the benefit of free time in the range of 300 ms to several seconds that we investigated here.

3. In the original model by Popov and Reder (2020), the resource taken by each encoding event is a fixed amount, which does not change with the available resources. Here, we changed it to a fixed proportion of the available resources.

4. The R script for the model described here and simulating the data is available at https://osf.io/egz64.
