# Supplementary material for: What Is Time Good for in Working Memory?
Source: Psychol Sci. 2021 Jul 26;32(8):1325–37. doi: 10.1177/0956797621996659 (PMC13038132; doi:10.1177/0956797621996659)
Supplement: sj-docx-1-pss-10.1177_0956797621996659 – Supplemental material for What Is Time Good for in Working Memory? [file sj-docx-1-pss-10.1177_0956797621996659.docx]

**Results**

**Temporal grouping effects in Experiment 2b**

We first analyzed response times to examine the effects of the short and long gap due to temporal grouping. Response times for the item after the gap should be longer compared to the no gap condition. We compared the response times to this critical item from short gap condition with no gap condition with a Bayesian t-test and found longer response times in the short gap condition (BF10: 66). We did the same analysis for the comparison of long gap condition and no gap condition and found the same effect (BF10: 275). We also compared the short and long gap conditions to understand whether the long gap condition had more impact and found no difference between the conditions in the response times given to this item (BF01: 2.66).

We then analyzed the serial recall accuracy differences between the conditions with Bayesian t-tests. Both the short gap condition (BF10: 91) and the long gap condition (BF10: 18) had higher serial recall accuracy compared to the no gap condition. We also visually examined the serial recall curves and observed the typical effects of temporal grouping: Small primacy and recency effects within each group on top of the overall serial-position effects (See Figure 1). These findings assured us that for both conditions, there were equivalent temporal grouping effects.


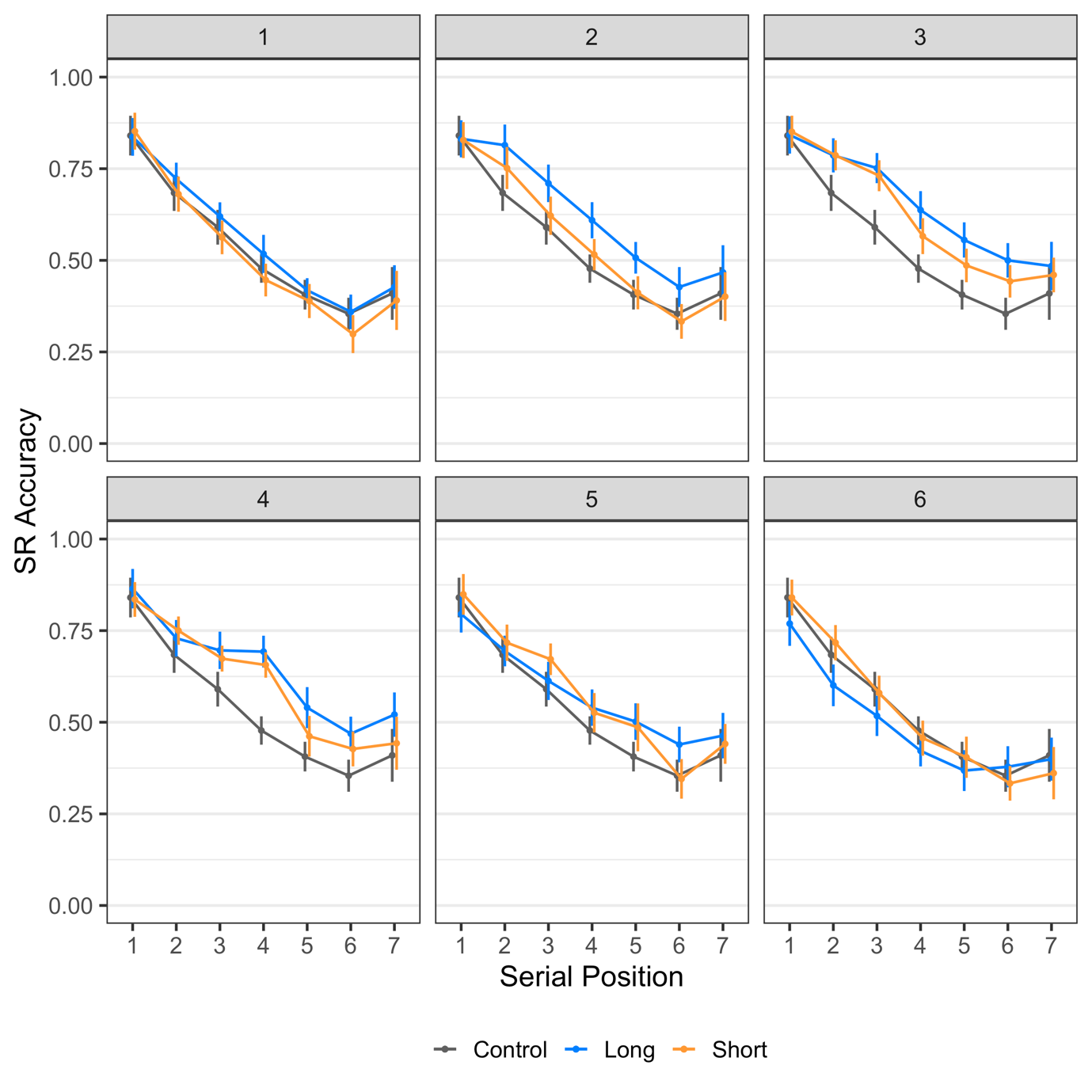


Figure S1. Serial position curves from Experiment 2b. Proportion correct of the immediate serial recall memory performance of the three conditions for each free time position and serial position. Panels correspond to the free time position (1 to 6). Error bars denote 95% within-subjects confidence intervals.

*
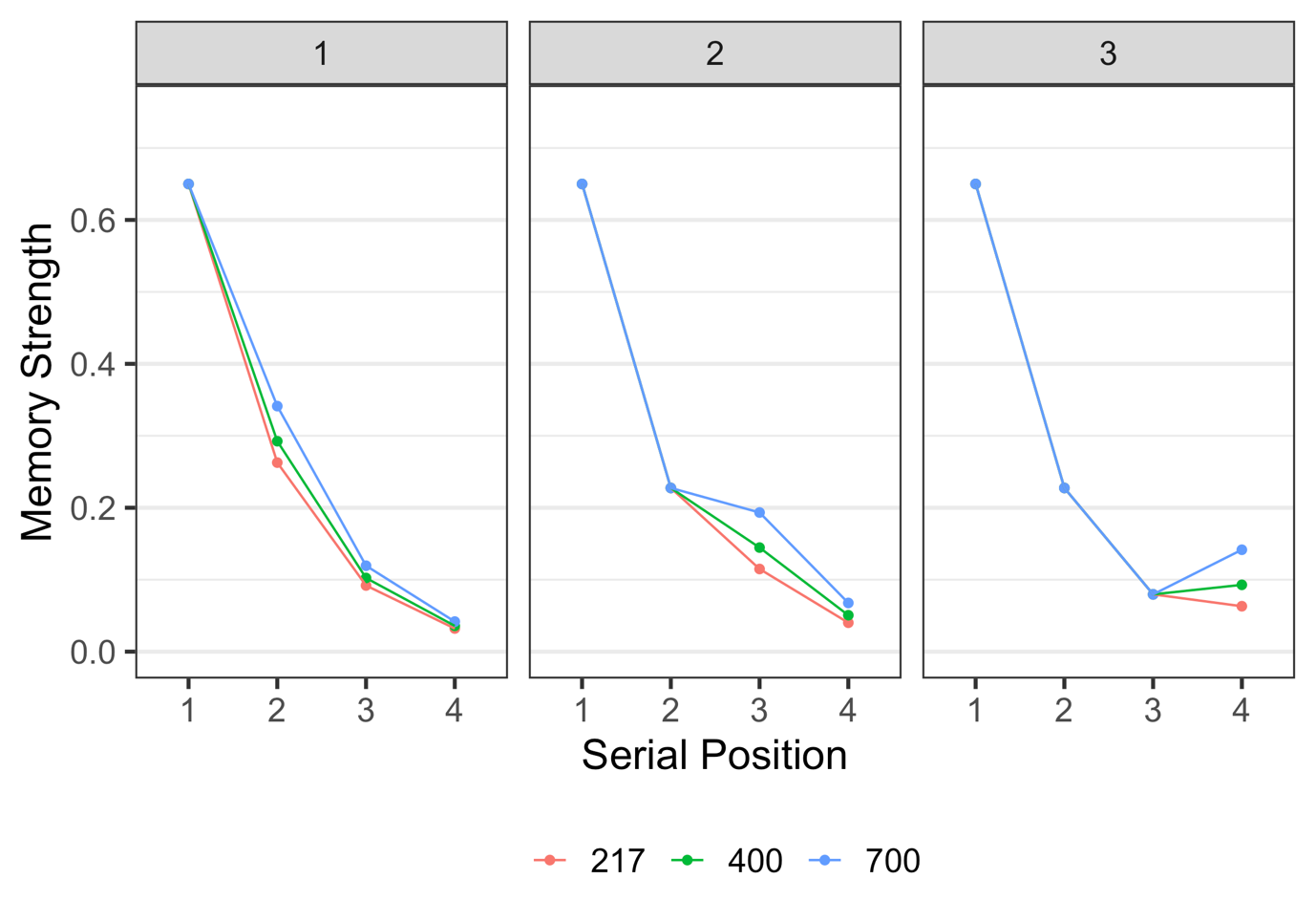
*

Figure S2. Predicted memory strength by the model for Ricker and Hardman (2016) Experiment 4. The panels show performance for three gap durations (217 ms, 400ms, and 700ms) at specific gap positions (1, 2, and 3) as a function of serial position. Parameter values used for this simulation are: *p* = 0.65, *r* = 0.25, *gain* = 15, *τ* = 0.11.

**Table S1. Bayes Factors and Log Bayes Factors for all the models for Experiment 2a analysis 1**

| Model | Effects | BF Random intecept only | Log BF | BF Maximal | Log BF |
| --- | --- | --- | --- | --- | --- |
| Model 1 | Free Time | 1.62E+00 | 0.482937 | 3.72E+02 | 5.919791 |
| Model 2 | Lag Sign | 4.79E+117 | 270.968207 | 2.41E+11 | 26.208468 |
| Model 3 | Abs_Lag | 1.82E-02 | -4.007968 | 2.19E+03 | 7.692458 |
| Model 4 | Free Time + Lag Sign | 2.71E+120 | 277.308841 | 1.07E+14 | 32.307359 |
| Model 5 | Abs_Lag + Lag Sign | 3.28E+117 | 270.589364 | 5.36E+14 | 33.915404 |
| Model 6 | Free Time + Abs_Lag | 3.01E-02 | -3.504477 | 7.59E+05 | 13.539342 |
| Model 7 | Free Time + Abs_Lag + Lag Sign | 2.30E+120 | 277.143326 | 1.85E+17 | 39.761259 |
| Model 8 | Free Time * Lag Sign | 2.75E+121 | 279.624313 | 4.98E+15 | 36.144273 |
| Model 9 | Abs_Lag * Lag Sign | 1.07E+169 | 389.201116 | 5.43E+45 | 105.307783 |
| Model 10 | Free Time * Abs_Lag | 3.56E-04 | -7.939395 | 2.14E+05 | 12.274202 |
| Model 11 | Free Time + (Lag Sign * Abs_Lag) | 4.08E+174 | 402.056392 | 1.92E+48 | 111.175036 |
| Model 12 | Lag Sign + (Free Time * Abs_Lag) | 3.80E+118 | 273.03955 | 5.17E+16 | 38.483745 |
| Model 13 | Abs_Lag + (Free Time * Lag Sign) | 2.50E+121 | 279.529824 | 1.16E+19 | 43.893463 |
| Model 14 | Model 7 + Free Time:Abs_Lag + Lag_Sign:Free Time | 4.22E+119 | 275.447478 | 3.07E+18 | 42.568776 |
| Model 15 | Model 7 + Free Time:Abs_Lag + Lag_Sign:Abs_Lag | 9.94E+172 | 398.341192 | 5.38E+47 | 109.903344 |
| Model 16 | Model 7 + Free Time:Lag_Sign + Lag Sign:Abs_Lag | 1.20E+177 | 407.741353 | 1.07E+50 | 115.196048 |
| Model 17 | Model 16 + Free Time:Abs_Lag | 3.05E+175 | 404.068186 | 3.02E+49 | 113.931858 |
| Model 18 | Free Time * Abs_Lag * Lag Sign | 1.05E+174 | 400.702661 | 2.86E+48 | 111.574954 |

**Table S1. Bayes Factors and Log Bayes Factors for all the models for Experiment 2b analysis 1**

| Model | Effects | BF Random intecept only | Log BF | BF Maximal | Log BF |
| --- | --- | --- | --- | --- | --- |
| Model 1 | Free Time | 3.04E-01 | -1.190987 | 2.51E+00 | 0.9207681 |
| Model 2 | Lag Sign | 1.34E+82 | 1.89E+02 | 3.73E+07 | 17.433842 |
| Model 3 | Abs_Lag | 1.79E-02 | -4.02E+00 | 2.77E+01 | 3.3230441 |
| Model 4 | Free Time + Lag Sign | 2.01E+82 | 1.90E+02 | 9.34E+07 | 18.3519157 |
| Model 5 | Abs_Lag + Lag Sign | 2.20E+81 | 1.87E+02 | 1.03E+09 | 20.7505348 |
| Model 6 | Free Time + Abs_Lag | 5.53E-03 | -5.20E+00 | 7.06E+01 | 4.2567901 |
| Model 7 | Free Time + Abs_Lag + Lag Sign | 3.46E+81 | 1.88E+02 | 2.61E+09 | 21.6819755 |
| Model 8 | Free Time * Lag Sign | 2.75E+86 | 1.99E+02 | 3.56E+11 | 26.597734 |
| Model 9 | Abs_Lag * Lag Sign | 8.83E+106 | 2.46E+02 | 3.34E+28 | 65.6786774 |
| Model 10 | Free Time * Abs_Lag | 1.38E-04 | -8.89E+00 | 9.01E+02 | 6.8032393 |
| Model 11 | Free Time + (Lag Sign * Abs_Lag) | 3.73E+107 | 2.48E+02 | 8.46E+28 | 66.6082566 |
| Model 12 | Lag Sign + (Free Time * Abs_Lag) | 2.72E+80 | 1.85E+02 | 3.27E+10 | 24.2113783 |
| Model 13 | Abs_Lag + (Free Time * Lag Sign) | 5.85E+85 | 1.97E+02 | 1.01E+13 | 29.9393826 |
| Model 14 | Model 7 + Free Time:Abs_Lag + Lag_Sign:Free Time | 5.11E+84 | 1.95E+02 | 1.30E+14 | 32.4983616 |
| Model 15 | Model 7 + Free Time:Abs_Lag + Lag_Sign:Abs_Lag | 5.95E+106 | 2.46E+02 | 1.05E+30 | 69.1244899 |
| Model 16 | Model 7 + Free Time:Lag_Sign + Lag Sign:Abs_Lag | 4.83E+113 | 2.62E+02 | 3.26E+32 | 74.8642492 |
| Model 17 | Model 16 + Free Time:Abs_Lag | 9.50E+112 | 2.60E+02 | 4.16E+33 | 77.4097588 |
| Model 18 | Free Time * Abs_Lag * Lag Sign | 3.95E+112 | 2.59E+02 | 2.06E+36 | 83.6158957 |
